# Efficacy and safety of the Ayurvedic herbal preparation Maharishi Amrit Kalash: a systematic review of randomized controlled trials

**DOI:** 10.3389/fmed.2024.1325037

**Published:** 2024-04-16

**Authors:** Anna K. Koch, Manish Patel, Shivenarain Gupta, Ricarda Wullenkord, Michael Jeitler, Christian S. Kessler

**Affiliations:** ^1^Department of Internal Medicine and Nature-Based Therapies, Immanuel Hospital Berlin, Berlin, Germany; ^2^Division of Oncology and Hematology, Department of Pediatrics, Charité Universitätsmedizin Berlin, Corporate member of Freie Universität Berlin and Humboldt-Universität zu Berlin, Berlin, Germany; ^3^Post Graduate Department of Kayacikitsa, J. S. Ayurveda College, Nadiad, India; ^4^Head of Academic Advisory Board, European Academy of Ayurveda, Birstein, Germany; ^5^Applied Social Psychology and Gender Research, CITEC, Bielefeld University, Bielefeld, Germany; ^6^Institute of Social Medicine, Epidemiology, and Health Economics, Charité Universitätsmedizin Berlin, Corporate Member of Freie Universität Berlin and Humboldt-Universität zu Berlin, Berlin, Germany

**Keywords:** Ayurveda, systematic review, traditional Indian medicine, herbal medicine, maharishi Amrit Kalash

## Abstract

**Background:**

Maharishi Amrit Kalash (MAK) 4 and 5 are Ayurvedic herbal nutritional supplements that are believed to have beneficial effects on overall health and wellbeing. This study aimed to systematically review all available randomized controlled trials (RCTs) investigating the clinical effects and safety of MAK.

**Methods:**

We included RCTs on therapy, health promotion, and prevention for patients and healthy volunteers of all ages. We systematically searched MEDLINE (via PubMed), EMBASE (via Ovid), the Cochrane Central Register of Controlled Trials (CENTRAL, The Cochrane Library), DHARA, Clinicaltrials.gov, the World Health Organization (WHO) International Clinical Trials Registry Platform, and Google Scholar from inception through 7 May 2023, with no time or language restrictions. The risk of bias was assessed using the Cochrane Risk of Bias Tool version 1. The protocol was registered with PROSPERO before conducting the review (CRD42023421655).

**Results:**

Three RCTs with 418 study participants were included. Two studies were on breast cancer patients and one on healthy adults. The two studies on cancer evaluated the efficacy of MAK in reducing the side effects of chemotherapy in women with breast cancer. The study on healthy adults evaluated whether MAK has an effect on an age-related alertness task as an indicator of cognitive aging. Both studies on breast cancer patients found beneficial effects on performance status, anorexia, vomiting, and body weight. One study reported positive effects regarding stomatitis. Regarding visual alertness, results showed that individuals who received MAK improved in performance. None of the three included studies reported adverse events. The risk of bias was mixed. Due to the small number and heterogeneity of the RCTs, no meta-analysis could be performed.

**Conclusion:**

There is evidence that MAK may have supportive effects in chemotherapeutic treatments for breast cancer patients and for healthy individuals regarding visual discrimination. However, it is difficult to verify treatment effects due to the small number of RCTs and the mixed risk of bias. Furthermore, none of the included studies recorded adverse events. Therefore, further high-quality studies are warranted to confirm the potential health benefits of MAK and to determine its optimal dosage and duration of use.

**Systematic review registration:**

PROSPERO, CRD42023421655.

## 1 Introduction

Ayurveda is a traditional system of medicine originating in South Asia and has been practiced for more than 2000 years on the Indian subcontinent and elsewhere. It is based on the belief that a person’s physical, mental, and spiritual wellbeing is dependent on an individual balance between the body, mind, and soul. It is recognized by the World Health Organization (WHO) and is widely practiced today, including in the Western world (1–3). There are guidelines for the clinical evaluation of Ayurvedic interventions to ensure quality in this area of research (4). Ayurveda includes a wide range of medical practices, such as individualized treatments consisting of manual therapies, purification treatments (“*Pancakarma*”), nutritional therapy and herbs, lifestyle counseling, and yoga exercises (5). Maharishi Ayurveda is a contemporary revival that takes into account these traditional approaches in agreement with the classical texts (6). Since 2014, Ayurveda in India has been regulated by an independent ministry (Ministry of Ayurveda, Yoga, Naturopathy, Unani, Siddha, Sowa-Rigpa and Homoeopathy; abbreviated as AYUSH Ministry (7)).

Maharishi Amrit Kalash (MAK) 4 and 5 are Ayurvedic herbal preparations that are believed to have beneficial effects on overall health and wellbeing. These preparations are combinations of several herbs and minerals with *rasayana* (rejuvenative and immune boosting) effects and is said to be helpful in supporting the body’s natural defenses against disease. MAK 4 is prepared as a paste, whereas MAK 5 is administered as tablet. The ingredients of the two delivery forms differ from each other, a phytochemical standardization of the preparations is being sought (8), see Table 1. The preparations for MAK 4 and MAK 5 are based on the classic Ayurvedic formulation for Brahma Rasayana, as described in traditional texts and the Ayurvedic Pharmacopoeia of India (9, 10). The MAK preparation is complex, with a range of pharmacological activities on various organ systems. In terms of its preparation, it is comparable to the classic Ayurvedic formulation Chyavanprash with regard to numerous ingredients (e.g., *Emblica officinalis*) for which mechanisms have been discussed (11, 12). Studies have shown that Chyavanprash has immunostimulatory effects, enhancing the secretion of cytokines and stimulating macrophage and natural killer cell activity (13). Based on similarities in the preparations and the range of indications, similar mechanisms of action can also be assumed for MAK. Both Chyavanprash and the classic Ayurvedic recipe Brahma Rasayana, on which MAK is based, are classified as *rasayana* in Ayurveda, aimed at maintaining vigor, vitality, and delaying the aging process (14). These mechanisms might contribute to its therapeutic potential for various health conditions. *In vitro* effects for MAK 4 and 5 have been shown in different studies. Inaba et al. (15) evaluated the immunomodulatory effects of MAK 4 and MAK 5 in mice. MAK 4 increased the responsiveness of lymphocytes, and MAK 5 increased not only the responsiveness of lymphocytes but also macrophage function. In this study, it is also suggested that MAK 4 and 5 have mitogenic effects on lymphocytes. Sugiura et al. (16) found that MAK 4 and 5 were found to promote the phagocytic and digestive functions of macrophages in mice compared with control and also had a stimulatory effect on macrophages. Furthermore, Penza et al. (17) found that a MAK-supplemented diet inhibited liver carcinogenesis in urethane-treated mice. Several but fewer studies have also investigated the *in vivo* effects of MAK 4 and 5. Sundaram et al. (18) treated 10 hyperlipidemic patients receiving stable hypolipidemic therapy with MAK 4 and 5 for 18 weeks. Plasma lipoprotein, plasma lipid peroxide, and low-density lipoprotein oxidation studies were evaluated every 6 weeks. The results indicate that MAK 4 and MAK 5 may be useful in the prevention and treatment of atherosclerosis. Zanella et al. (19) put healthy people on diets with or without MAK and found that a MAK-enriched diet reduced oxidative stress parameters and increased antioxidant defenses in both short- and long-term treatment. Accordingly, there is quite some evidence that MAK may have positive effects on various health parameters. However, a systematic review of the available evidence is still lacking.

**Table 1:** Preparation of Maharishi Amrit Kalash Paste (MAK 4) (9, 10).

**Name of ingredient**		**Part used**	**Composition in mg /20 gm**
**Latin**	**English**		
*Saccharum officinarum*	Sugarcane	Sugar	12,400,00
Aqua	Water		3,850,00
	Ghee		500,00
*Emblica officinalis* (Organic)	Indian gooseberry	Fruit	910,00
Mel	Honey		800,00
*Terminalia chebula* (Organic)	Chebulic myrobalan	Fruit	455,00
*Centella asiatica* (Organic)	Indian penny wort	Whole plant	78,00
*Cinnamomum zeylanicum* (Organic)	Cinnamon	Bark	78,00
*Convolvulus pluricaulis* (Organic)	Aloeweed	Whole plant	78,00
*Curcuma longa* (Organic)	Turmeric	Rhizome	78,00
*Cyperus rotundus* (Organic)	Nutgrass	Root	78,00
*Cyperus scariosus* (Organic)	Nutgrass	Tuberous root	78,00
*Elettaria cardamomum* (Organic)	Lesser cardamom	Fruit	78,00
*Embelia ribes* (Organic)	Butterfly pea	Fruit	78,00
*Glycyrrhiza glabra* (Organic)	Liquorice	Root	78,00
*Mesua ferrea* (Organic)	Cobra’s saffron	Flower	78,00
*Piper longum* (Organic)	Long pepper	Fruit	78,00
*Santalum album*	Sandalwood White	Heartwood	78,00
*Polygonatum verticillatum* (Aqueous Extract)	-	Root	31,00
*Asparagus racemosus* (Organic) (Aqueous Extract)	Indian asparagus	Root	21,40
*Boerhavia diffusa* (Organic) (Aqueous Extract)	Spreading hogweed	Whole plant	12,60
*Oroxylum indicum* (Organic) (Aqueous Extract)	Indian trumpet tree	Bark	7,60
*Solanum xanthocarpum* (Organic) (Aqueous Extract)	Yellow berried nightshade	Whole plant	7,20
*Gmelina arborea* (Organic) (Aqueous Extract)	Cashmere tree	Bark	6,80
*Pueraria tuberosa* (Organic) (Aqueous Extract)	Indian kudzu	Tuberous root	5,80
*Teramnus labialis* (Organic) (Aqueous Extract)	-	Whole plant	5,80
*Tribulus terrestris* (Organic) (Aqueous Extract)	Small caltrops	Fruit	5,80
*Ipomoea digitata* (Organic) (Aqueous Extract)	Giant potato	Tuberous root	5,40
*Uraria picta* (Organic) (Aqueous Extract)	-	Whole plant	5,40
*Clerodendrum phlomidis* (Organic) (Aqueous Extract)	-	Whole plant	4,40
*Sida cordifolia* (Organic) (Aqueous Extract)	Country mallow	Whole plant	4,40
*Solanum indicum* (Organic) (Aqueous Extract)	Indian nightshade	Whole plant	3,60
*Leptadenia reticulata* (Organic) (Aqueous Extract)	-	Whole plant	3,20
*Phaseolus trilobus* (Organic) (Aqueous Extract)	Wild gram	Whole plant	3,20
*Stereospermum suaveolens* (Organic) (Aqueous Extract)	Yellow snake tree	Bark	3,20
*Desmodium gangeticum* (Organic) (Aqueous Extract)	Tick trefoil	Whole plant	2,80
*Pedalium murex* (Organic) (Aqueous Extract)	Large caltrops	Fruit	2,80
*Saccharum spontaneum* (Aqueous Extract)	Thatch grass	Root	2,20
*Eragrostis cynosuroides* (Aqueous Extract)	Feather grass	Root	1,80
*Saccharum officinarum* (Aqueous Extract)	Sugarcane	Root	1,40
*Aegle marmelos* (Organic) (Aqueous Extract)	Bael	Bark	1,20

The preclinical evidence for MAK is already well-reviewed, but the necessary clinical evidence still largely lacking. To date, there has been no systematic review that assesses and compares the efficacy and safety of MAK for the prevention and treatment of various health conditions and in healthy individuals. The aim of this review is to summarize the existing randomized controlled trials (RCTs) on the efficacy and safety of MAK, to provide a comprehensive overview of the existing clinical evidence on MAK and to identify areas where further prospective clinical research is required.

## 2 Methods

This systematic review was conducted and reported in accordance with the Preferred Reporting Items for Systematic Reviews and Meta-Analyses (PRISMA) guidelines (20, 21). The protocol was registered with PROSPERO before conducting the review (CRD42023421655).

### 2.1 Eligibility assessment

Randomized controlled, randomized crossover, and cluster randomized trials were eligible. Studies of individuals of any age, sex, and origin were included. We included trials of therapeutic, health-promoting, or preventive use of MAK. Studies that compared MAK with (1) no specific intervention, (2) placebo, (3) other medicine treatment, or (4) other Ayurvedic preparations were eligible. Studies that examined MAK in combination with other procedures were included only if the concurrent intervention was comparable between all groups. There were no restrictions on the type of outcomes.

### 2.2 Search strategy and databases

MEDLINE (via PubMed), EMBASE (via Ovid), Cochrane Central Register of Controlled Trials (CENTRAL, The Cochrane Library), DHARA, Clinicaltrials.gov, the WHO International Clinical Trials Registry Platform were searched without time and language restrictions from inception through 7 May 2023. In order to include gray literature (e.g., reports, government documents, dissertations, theses, and conference abstracts), Google Scholar was also searched. The complete search strategy for PubMed is shown in Table 2. Search strategies for the other databases were identical in content except for the fact that we did not filter for RCTs for Google Scholar but included all hits for “Maharishi Amrit Kalash” for a maximum sensitive gray literature search.

**Table 2:** Search strategy for MEDLINE (via PubMed) using the Cochrane Highly Sensitive Search Strategy for identifying randomized trials in MEDLINE: sensitivity-maximizing version (2008 revision); PubMed format (22).

#1	randomized controlled trial [pt]
#2	controlled clinical trial [pt]
#3	randomized [tiab]
#4	placebo [tiab]
#5	drug therapy [sh]
#6	randomly [tiab]
#7	trial [tiab]
#8	groups [tiab]
#9	#1 OR #2 OR #3 OR #4 OR #5 OR #6 OR #7 OR #8
#10	animals [mh] NOT humans [mh]
#11	#9 NOT #10
#12	“Maharishi Amrit Kalash” or “MAK 5” or “MAK5” or “MAK 4” or “MAK4”
#13	#11 AND #12

### 2.3 Study selection and data extraction

Search results were checked for duplicates using the open-source software rayyan.ai. Two authors (AKK and RW) independently screened abstracts and full texts for eligibility using rayyan.ai. Disagreements were resolved in discussion with a third author (CK) until a consensus was reached. Study characteristics were extracted using a pre-developed data extraction form independently by two authors (AKK and RW). Data on publication type, design and funding, participants, intervention arms, dosage and pharmaceutical form, outcomes, and safety were extracted from the included full texts.

### 2.4 Risk of bias of individual studies

Two authors (AKK and RW) independently assessed the risk of selection bias, performance bias, detection bias, attrition bias, reporting bias, and other biases using the Cochrane Risk of Bias tool version 1 (23). Disagreements were resolved in discussion with a third author (CK) until a consensus was reached.

### 2.5 Data synthesis

If at least two studies assessing this specific outcome are available, meta-analyses were planned to be conducted using the Statistical Package for Social Sciences software (IBM SPSS Statistics for Windows, release 29.0; IBM Corporation, Armonk, NY) by a random effects model. Mean differences (MDs) between groups and their 95% confidence intervals (CIs) would have been calculated. The effects of MAK compared with different control interventions were planned to be analyzed separately. In case of data missing, attempts would have been made to obtain the missing data from the trial authors by email. Ultimately, pairwise meta-analyses could not be performed because of the small number of included studies.

### 2.6 Assessment of statistical heterogeneity

Statistical heterogeneity was planned to be evaluated using chi-square (*χ*^2^) statistics with a *p*-value of ≤0.10, indicating significant heterogeneity. The extent of heterogeneity was categorized using *I*^2^, with *I*^2^ > 25% representing moderate, *I*^2^ > 50% representing substantial, and *I*^2^ > 75% representing considerable heterogeneity (24). Ultimately, the assessment of statistical heterogeneity could not be performed because of the small number and poor reporting of included studies.

### 2.7 Subgroup analyses

The following subgroup analyses were planned *a priori*: If studies on both (1) MAK 4 and MAK 5 or (2) participants older and younger than 18 years were found, they would be considered separately. Due to the small number of included studies, none of the planned subgroup analyses could be performed.

## 3 Results

### 3.1 Literature search

**Figure 1:**
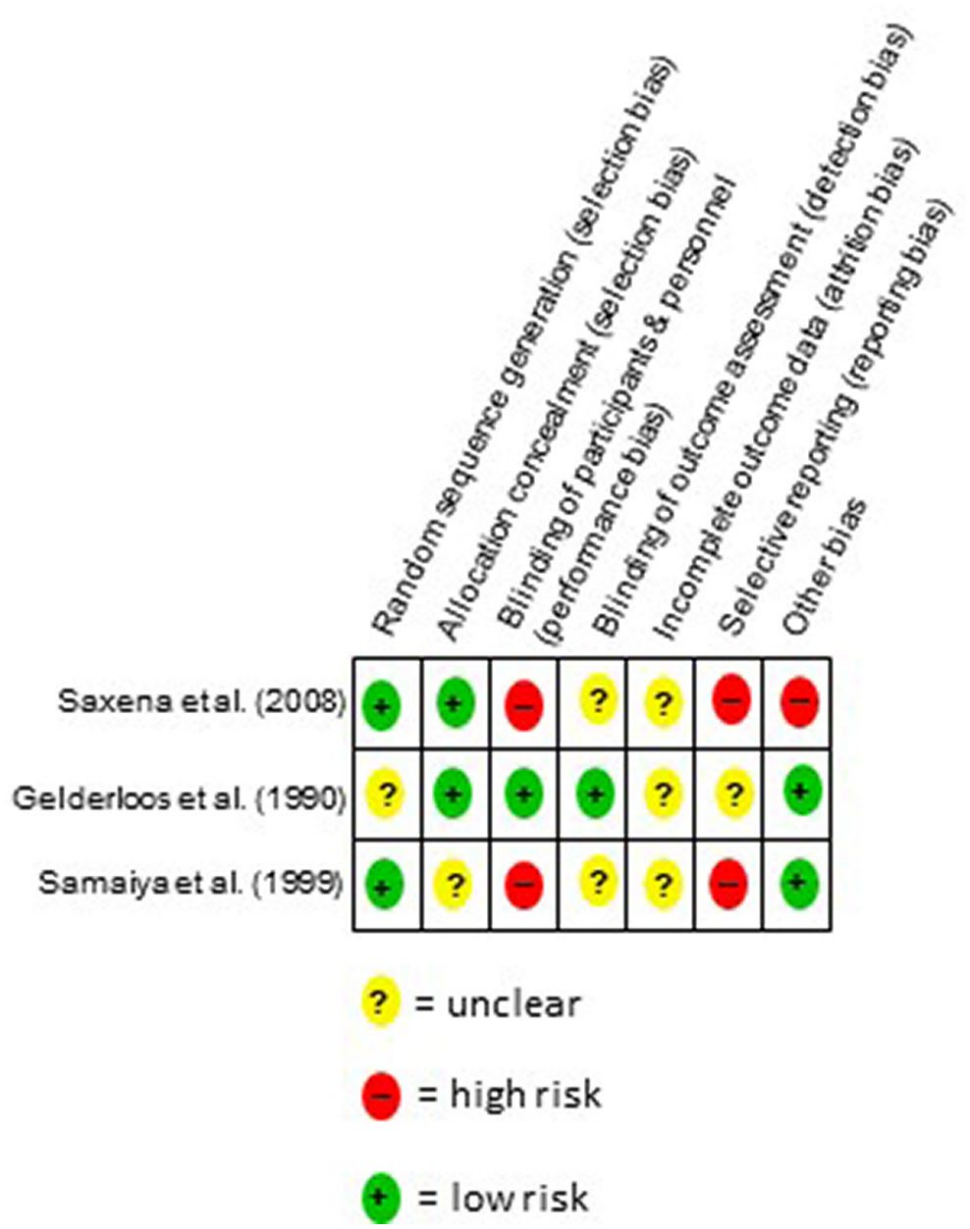
Flow chart of the study selection process.

The literature search revealed 575 records; the identification of studies on other methods yielded 12 additional records (Figure 1). After excluding duplicates and irrelevant abstracts, 20 full texts were identified to be assessed for eligibility. For six of those studies, no full text could be retrieved at the time of analysis: Two study registries (25, 26), of which one has been published in the mean time (25) three conference abstracts (27–29), and one dissertation (30). Eight full texts were excluded because there were no RCT (18, 31–37), and three because it was the wrong intervention (37–39). Hence, three RCTs (*N* = 418) were included in the systematic review (40–42).

### 3.2 Study characteristics

Study characteristics are presented in detail in Table 3. Two studies examined breast cancer patients (41, 42), and one focused on healthy adults (40). The two studies on cancer evaluated the efficacy of MAK in reducing the side effects of chemotherapy in women with breast cancer. The underlying hypothesis was that since MAK is rich in antioxidants, a reduction in the toxicity of chemotherapy can be achieved. The study on healthy adults evaluated whether MAK has an effect on an age-related alertness task. The underlying hypothesis was that MAK positively affects attentional capacity or alertness and thus can reverse the cognitive effects of aging. The studies were carried out in India (41, 42) and the USA (40). All studies were published between 1990 and 2008 and used a randomized study design. One study compared MAK to placebo (40), and the other two studies compared chemotherapy plus MAK to chemotherapy alone (41, 42). The sample size varied between *n* = 60 and *n* = 214 participants.

**Table 3:** Study characteristics.

Study	Publication type	Design and funding	Participants	Intervention arms	MAK dosage and pharmaceutical form	Outcomes	Significant group differences	Safety
Gelderloos et al. (1990)	Journal article	RCT Origin: USA Funding: n.a.	Healthy adults older than 35 years Randomized: *N* = 60 Analyzed: *n* = 48 (*n* = 22 experimental, *n* = 26 control) Gender: 100% male Mean age ± SD: 39.9 ± 2.86 experimental; 39.4 ± 3.99 control	Experimental: MAK Control: Placebo; after the study the control group also received MAK	One MAK tablet twice daily for 6 weeks	Performance as measured by visual discrimination: Whole field and three subfields (A, B, C) at three time points: (T1) before treatment, (T2) after 3 weeks of treatment, (T3) after 6 weeks of treatment	Whole field and field A at weeks 3 and 6 in favor of MAK	n.a.
Samaiya et al. (1999)	Journal article	RCT Origin: India Funding: Maharshi Ayurveda Products	Breast cancer patients receiving chemotherapy Randomized: *N* = 129 (*n* = 61 experimental, *n* = 68 control) Analyzed: *n* = 112 (*n* = 53 experimental, *n* = 59 control) Gender: n.a. Mean age ± SD: 43.33 (SD not reported) experimental; 45.4 (SD not reported) control	Experimental: CMF or CAF plus ondansetron + MAK Control: CMF or CAF plus ondansetron	1 tablespoon (10gm) MAK 4 paste twice daily with milk and 1 tablet MAK 5 twice daily with water during the entire period of chemo	Toxicity of anticancer chemotherapy as measured by side effects: general well-being, anorexia, performance status as measured with the KPS, leucopoenia, stomatitis, vomiting, diarrhea, alopecia, fever, allergy, pulmonary and neurotoxicity, cardiotoxicity, cutaneous manifestation, pain, weight Tumor response Outcomes assessed at baseline and 6 cycles of chemotherapy	KPS after fourth cycle in favor of MAK Anorexia after third cycle in favor of MAK Vomiting after third and fourth cycle in favor of MAK Stomatitis after fourth cycle in favor of MAK Weight in favor of MAK	n.a.
Saxena et al. (2008)	Journal article	RCT Origin: India Funding: Maharshi Ayurveda Products	Female breast cancer patients receiving chemotherapy Randomized: *N* = 214 Analyzed: *n* = 181 (*n* = 102 experimental, *n* = 112 control) Gender: 100% female. Mean age ± SD: 44 ± 10 experimental; 44.9 ± 8.9 control	Experimental: CMF or CAF plus ondansetron and dexamethasone + MAK Control: CMF or CAF plus ondansetron and dexamethasone	2 tablespoons MAK 4 paste twice daily with a glass of milk and 2 tablets MAK 5 twice daily with lukewarm water half an hour after MAK 4, for approximately 18 weeks	Toxicity of anticancer chemotherapy as measured by side effects: anorexia, vomiting, stomatitis, diarrhea, alopecia, performance status as measured with the KPS, weight, and leucopoenia Tumor response Outcomes assessed at baseline and 6 cycles of chemotherapy	Anorexia in all cycles, however differences were only clearly reported in the fourth cycle in favor of MAK Vomiting in third and fourth cycle in favor of MAK KPS in fifth cycle in favor of MAK Weight in favor of MAK	n.a.

RCT, randomized controlled trial; n.a., not available; SD, standard deviation; MAK, Maharishi Amrit Kalash; CMF, Cyclophosphamide, methotrexate and 5-flourouracil; CAF, cyclophosphamide, adriamycin, and 5-flourouracil; KPS, Karnofsky performance scale.

### 3.3 Study findings

#### 3.3.1 MAK dosage and pharmaceutical form

Gelderloos et al. (40) administered one MAK tablet twice daily for 6 weeks. Samaiya et al. (41) administered 1 tablespoon (10gm) MAK 4 paste twice daily with milk and one tablet MAK 5 twice daily with water during the entire period of chemotherapy. Saxena et al. (42) administered 2 tablespoons MAK 4 paste twice daily with a glass of milk and two tablets MAK 5 twice daily with lukewarm water half an hour after MAK 4, for approximately 18 weeks.

#### 3.3.2 MAK as a supplement to chemotherapy in breast cancer

Both studies on breast cancer patients (41, 42) assessed outcomes at baseline and at each of the 6 cycles of chemotherapy. Both studies found a positive effect of MAK on *performance status* as measured with the Karnofsky performance scale (43). Samaiya et al. (41) after the fourth chemotherapy cycle, and Saxena et al. (42) after the fifth cycle in favor of MAK. Saxena et al. (42) found positive effects of MAK in *anorexia* in all cycles; however, differences were only clearly reported in the fourth cycle in favor of MAK. Samaiya et al. (41) reported positive effects on *anorexia* after the third cycle in favor of MAK. Regarding *vomiting*, both studies found positive effects of MAK after the third and fourth cycles. Both studies found positive effects on *body weight*. Furthermore, Samaiya et al. (41) reported positive effects regarding *stomatitis* after the fourth cycle in favor of MAK.

#### 3.3.3 MAK for reversing cognitive effects of aging

Gelderloos, Ahlstrom, Orme-Johnson, Robinson, Wallace, and Glaser (40) used a *visual alertness task* as an indicator of cognitive aging. Outcomes were assessed at three time points: before treatment with MAK, after 3 weeks of treatment, and after 6 weeks of treatment. Results showed that individuals who received MAK improved in performance on two of four measured fields at weeks 3 and 6.

#### 3.3.4 Safety

None of the included studies recorded adverse events.

### 3.4 Assessment of the scope of unpublished data

During the literature search, 181 studies were identified via clinicaltrials.gov and the WHO International Clinical Trials Registry Platform. Of these, two were potentially suitable (25, 26). The study titled “Effects of Herbal Antioxidants on Cardiovascular Disease in Older Blacks” was updated as “completed” with a final update in 2010 (26). However, results were not filed and could not be identified during the literature search, so a final assessment was not possible. The study titled “Role of MAK, Ayurvedic herbal medicine on Breast Cancer” was registered in 2019, and results are not yet available (25). In addition, the literature search identified three conference abstracts (27–29) and one dissertation (30) that could potentially be considered. However, no full-text publication could be found or retrieved for these. No further information could be obtained by writing to the authors either. Thus, there is a restriction with respect to the conclusion due to the scope of unpublished data.

### 3.5 Risk of bias of individual studies

**Figure 2:**
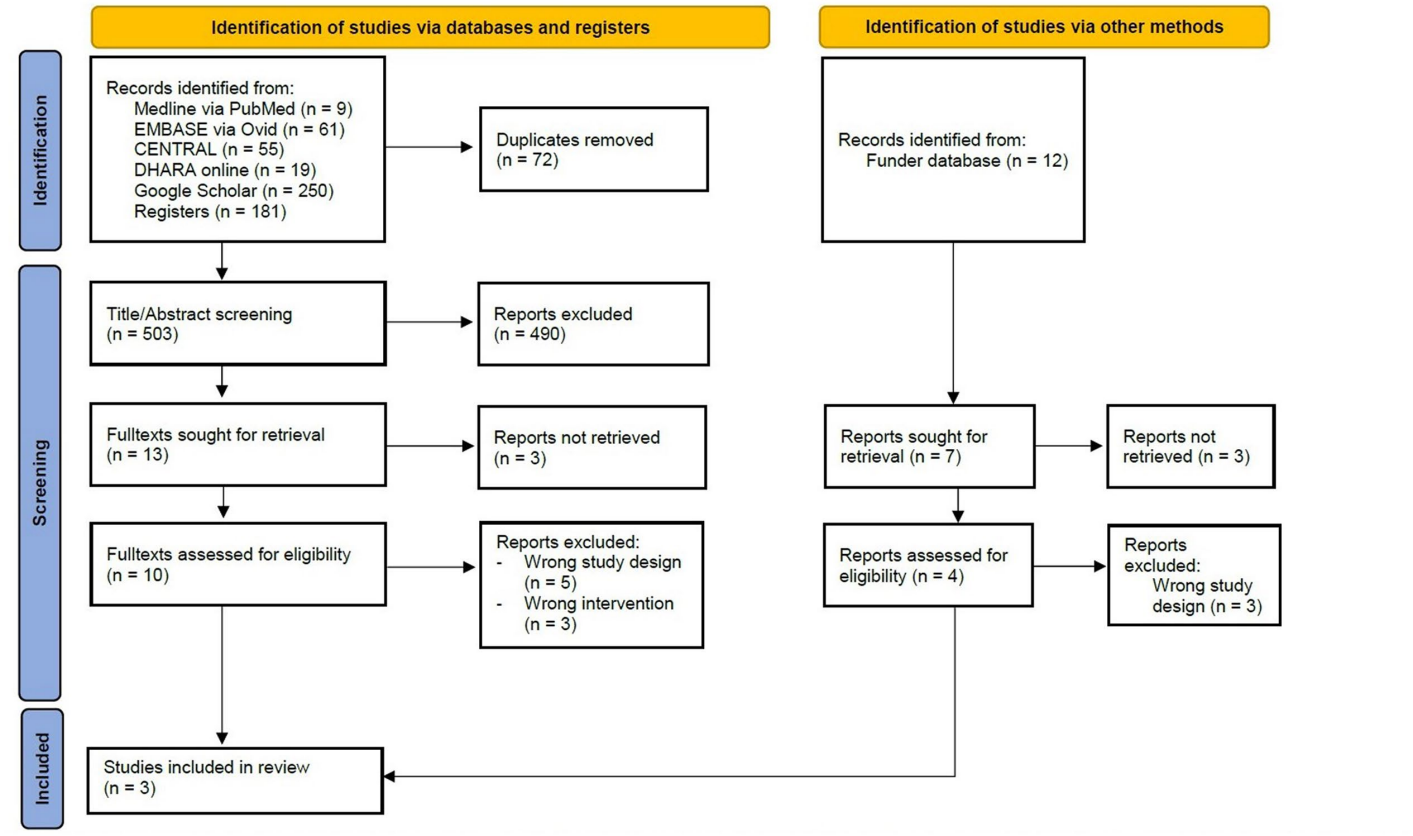
Risk of bias as assessed by the Cochrane Risk of Bias tool version 1.

The risk of bias was highly variable across both studies and domains (Figure 2). The risk of selection bias was low for Saxena et al. (42) and mixed for Samaiya et al. (41) and Gelderloos, Ahlstrom, Orme-Johnson, Robinson, Wallace, and Glaser (40). In Saxena et al. (42) and Samaiya et al. (41), a high bias was observed with regard to the blindings of participants and personnel, while for Gelderloos et al. (40) blinding was rated as adequate. Detection bias was rated low for Gelderloos et al. (40) and unclear for Saxena et al. (42) and Samaiya et al. (41). Attrition bias was rated unclear for all studies. The risk of bias with regard to reporting bias in Saxena et al. (42) and Samaiya et al. (41) was rated as high, while for Gelderloos et al. (40) it was rated as unclear. Other bias were considered low in Gelderloss et al. (40) and Samaiya et al. (41) and high in Saxena et al. (42).

## 4 Discussion

### 4.1 Summary of evidence

This systematic review provides new evidence as it is the first systematic review on MAK. It is based on three RCTs that included 418 participants in total (40–42). The results suggest that MAK may alleviate the side effects of chemotherapy in breast cancer patients and may positively influence attentional capacity or alertness in healthy adults. The results from these few RCTs complement the findings from other clinical trials suggesting beneficial effects of MAK. A recent scoping review concluded that preclinical studies show promising results for the use of MAK as an anticancer and chemoprotective agent (25). Furthermore, the results of Zanella et al. (19), who placed healthy individuals on a diet with or without MAK, showed that a MAK-enriched diet decreased oxidative stress parameters and increased antioxidant defenses in both short- and long-term treatments (12). Research into the phytochemical aspects of the plants that form the basis for the production of MAK has also shown promising prospects for the treatment of oxidative stress and cancer (45). Experiments in the mouse model also show positive effects of MAK on cancer-associated parameters—although here an effect on tumor incidence but not on body weight was shown (15–17). This is in contrast to the results of the two RCTs (41, 42), which showed positive effects of MAK on body weight but not on tumor response. The effects on cognitive attention parameters found by Gelderloos et al. (40) are complemented by findings from Nidich, Morehead, Nidich, Sands, and Sharma (39). In this study, the non-verbal intelligence of students who received a Maharishi Student Rasayana Food Supplement over a longer period of time within an RCT was compared. The results show an increase in non-verbal intelligence in the MAK group compared to the placebo. However, further research is urgently needed to prove these effects with regard to the effect of MAK on the side effects of chemotherapy and cognitive aging processes. Traditional, complementary and integrative medicine offers a variety of approaches to alleviate symptoms associated with some of today’s most pressing medical conditions, such as cancer, pain, and bowel disease, through procedures such as lifestyle changes and manual medicine (46, 47). Together with other procedures from traditional and complementary medicine, MAK might be a promising therapeutic addition.

### 4.2 Strengths and limitations

To our knowledge, this is the first systematic review of the therapeutic, health-promoting, and preventive effects of MAK without time or language limitations using a broad search strategy. This included searching clinical trial registries as well as gray literature. The results of the review indicate that there is a paucity of high-quality RCTs on this topic. RCTs on Ayurveda for common medical conditions are mostly scarce (48). Only three RCTs could be included in the present review, which substantially limits the strength of the evidence. The included studies showed a mixed risk of bias. Furthermore, bias due to unpublished data cannot be ruled out. For example, three conference abstracts could not be retrieved as full texts and consequently could not be included in the review (27–29). Furthermore, one potentially eligible study (26), registered on clinicaltrials.gov and categorized as completed with results, could not be retrieved, including results as well. Furthermore, methodological limitations may apply as well. Within the two studies on breast cancer (41, 42), it is not apparent whether the statistical analysis corrected for multiple testing when testing for differences in each chemotherapy cycle. The presentation of the results and the description in the text cast serious doubt on this, which further calls into question the validity of the results. The lack of *a priori* registrations in public study registries, the lack of recording of adverse events, and the lack of mention of defined primary and secondary outcome parameters in all three included studies (40–42) also deserve critical mention. Most of the studies are also relatively old. More recent studies based on current quality guidelines are urgently needed. Finally, it does not become clear from the study on healthy adults which MAK preparation is used (40). It is assumed that it is MAK 5, but this is not explicitly mentioned.

### 4.3 Clinical implications

Based on the available results, it is too early to make specific clinical implications. As with all dietary supplements, caution is advised when using them in a clinical context due to potential interaction effects with medications. It is also very important in this context that doctors and patients talk openly about the potential use of such supplements.

### 4.4 Implications for future research

Given that Ayurveda is not only widely practiced in South Asia but has become increasingly popular on a global scale (1, 2), there is a great need for high-quality RCTs to improve the quality of evidence for the effects and safety of MAK. Future RCTs should adhere to the established Ayurveda research quality standards (4) as well as take into account the international quality standards for RCTs, such as the Consolidated Standards of Reporting Trials (49, 50). There is also an urgent need for a structured recording and reporting of adverse events.

### 4.5 Conclusion

MAK 4 and 5 exhibit potential health benefits *in vivo*, but limited clinical RCTs and a high risk of bias complicate confirming treatment effects. Consultation with the treating physician is necessary, especially when supplementing conventional oncological therapy. High-quality studies are required to confirm MAK’s health benefits and to establish optimal dosage and intake duration.

## Data availability statement

The original contributions presented in the study are included in the article/supplementary material, further inquiries can be directed to the corresponding author.

## Author contributions

AK: Conceptualization, Data curation, Formal analysis, Methodology, Project administration, Validation, Visualization, Writing – original draft. MP: Supervision, Writing – review & editing. SG: Supervision, Writing – review & editing. RW: Data curation, Formal analysis, Investigation, Methodology, Writing – original draft. MJ: Supervision, Writing – review & editing. CK: Conceptualization, Funding acquisition, Supervision, Writing – review & editing.
